# The Golem of Psychology and the Ecosystemic Epistemology

**DOI:** 10.1007/s12124-020-09532-5

**Published:** 2020-05-01

**Authors:** Luca Tateo

**Affiliations:** University of Oslo, Oslo, Norway

**Keywords:** Ecological epistemology, Cultural psychology, Cogenetic logic, Epistemology of psychology

## Abstract

The old controversy about the epistemic status of psychological sciences is useless and sterile. Psychology is ether a hard science nor a soft science. It is an ecological life science, whose object is the whole system of co-developmental relations constituted by the presence of the organism in a given environment. After criticizing the positions of the traditional epistemic debate between hard and soft views of psychology, I propose a way to fundament the core concepts in the method of complementary negation, based on cogenetic logic. Then, I defend the need to develop a third way: an ecological epistemology of psychology.

## Introduction

Is psychology a pre-paradigmatic or a soft science (Zagaria et al. 2020)? Maybe yes and we can live with it. The epistemic status of the study of human activities and thinking has always been controversial (Koch and Leary 1992; Tateo 2013; Valsiner 2012). It seems that psychology is doomed to be “caught in the middle” between *Naturwissenschaften* and *Geisteswissenschaften* (Valsiner 2012, p. 137). The early modern formulation of this controversy can be traced back to the 17th Century’s criticism of Giambattista Vico to Descartes (Tateo 2017). Ever since, the terms of the debate have not really changed and the character of psychology as “atypical science” (Zagaria et al. 2020, p.) with respect to both its object and its method is still a major concern of psychologists playing the role of hard-scientists (Koch and Leary 1992). Zagaria et al. (2020) not surprisingly claim that psychological sciences are heterogeneous, ill-defined in both their object and their core concepts. The epistemic status of psychology as a science is not only a matter of internal coherence or cumulative knowledge, but also a matter of socio-historical conditions of its development, and of the social status of psychology as science of control (Valsiner 2012). Labelling psychology as “hard” or “soft” science would make a little difference for psychological knowledge about human beings, but would make a huge difference for the social credibility of psychologists and their access to economic and social resources. According to Zagaria et al. (2020), even the main object of psychology is uncertain. Whilst psychological sciences evoke the concept of “soul” in their name, the most part of current research would focus on “brain”, “mind” or “behavior”. The “soul” has been expelled as unscientific remnant of Christian theology, but it is rather an example of the scarce capacity of psychology to have a fruitful dialogue with the history of ideas, and with its own history in particular. As Valsiner (2012) has adamantly explained, psychology is flattened on its present or immediate past, often re-discovering ideas only because many relevant authors have been forgotten or misunderstood.

Looking just beyond the limited horizon of Western tradition, one could realize that in ancient Greek cosmology, Psyche (in Greek *psukhé*) is literally a “breath”, made of air that is capable to produce sounds and words by blowing out through the “windpipes” of the human body. The voice is literally the agent of the Psyche over the body in order to make the latter produce “sounds that mean something” to other people (Tateo 2019). The original meaning of Psyche is closer to the current understandings of psychic life developed, for instance, in Dialogical Self Theory (Hermans 2002) or in the embodied perspectives (Ingold 2000), which relate the sociogenesis of psychic functions, through internalization of social voices, with overcoming of the dualistic mind-body perspective. Another old controversy is the *mechanistic versus vitalistic* perspective in psychology, that is somehow echoed by the perspectives of neuropsychology and positive psychology. By eventually rejecting the vitalistic conception of the soul (Angell 1911), scientific psychology turned towards the mechanistic and analytical-summative view (Von Bertalanffy 1952) that is well represented by the behavioristics’ conception (Watson 1928). However, the dualistic view is also rooted in an ancient tradition that can be dated back to the Old Testament. In the *Psalm 139:16* one can find for the first time the term *galmi* (my golem) referring to a shapeless material or an embryo, before God inflating the breath of life. Also Adam, the first man, is called *golem* before he receives a soul (Gelbin 2011). This is the origin of the 14th Century image of an artificial man made of clay, who can be brought to life through an esoteric formula, widely known as *golem*. Still, contemporary psychology is stuck in the dichotomy between the idea of a mechanic explanation of low-level processes in an animal-machine and the idea of a ghost-in-the-machine individuality as instantiation of a kind of genetic or computational program (Ingold 2000). The golem of psychology is evoked in defense anytime a non-reductionist view of human psyche is proposed. This golem operates through the rhetoric devices of the “scientific” psychology (Valsiner 2012) and the positive psychology. Apparently opposite, the two rhetorics lead to a reductionist view of human psyche. The scientific psychology defends a summative, analytical, mechanistic low-level of explanation, based on the overproduction of evidence. In this case, reductionism is caused by the loss of the whole, both in the sense of the hyper-fragmentation of phenomena and concepts and in the loss of a theoretical development.

“Science is not a mere accumulation of facts; facts become knowledge only when incorporated into a conceptual system [.] The way of Science is from ideal cases, for which simple law can be enunciated, to the progressive inclusion of complications. [.] we know not too few but too many facts and that the very accumulation of an enormous amount of data hampers the discovery of the necessary theoretical schemes.” (Von Bertalanffy 1952, pp. 70–71).

Zagaria et al. (2020) do not escape this reductionism when they point out that the problem with the epistemic development of psychology is the lack of common definition between the core concepts. They do not consider that the problem may be the lack of theory to describe the *relationships* between the lower level concepts (Valsiner 2014). Using an analogy, one can say that there are three possible perspectives when one wants to know the functioning of a car engine. One can have the original project (metaphysical perspective); one can disassemble the engine in its smallest parts (analytical summative perspective); or one can observe the engine while functioning into a car when someone is driving (ecological perspective). Only the last perspective applies to human psyche, as the metaphysical one does not apply to an historically situated event and one cannot analytically disassemble a mind and still pretend to observe its functioning at the same time. The ecological perspective implies the understanding of the phenomenon in its development through a network of temporal and spatial relationships with something else. The epistemology discussed by Zagaria et al. (2020) is not a processual and systemic one. Neither is systemic the perspective of positive psychology (Lopez and Snyder 2009), which is reductionist in the sense that the spatio-temporal unfolding of psyche is flattened on the *here and now* of the *individual* taken out of its ecosystem. Miller (2008) notes: “instead of considering the social and political measures that might really improve people’s circumstances, positive psychology offers a substitute recipe for success, achievement and happiness that ultimately has no substance at all.” (Miller 2008).

Both scientific and positive psychology are not-developmental and potentially politically dangerous, to the extent that they do not focus on change and critique of the society but on the individual dimension of adaptive change. The former by paying attention to partial and instrumental dimensions (e.g. problem-solving, memory, etc.), the latter by putting the burden of change (or failure) only onto the individual (e.g. strength, character, etc.).

In its effort to overcome the reductionist tendencies, psychology has instead the potential to develop a third way to a non-reductionist and fully developmental epistemology. Psychology is definitely a life science, not in the sense that is akin to biology or genetics, but in the sense that: (a) deals with living organisms (stones do not have psychic life); (b) its object is historical (psychic life changes over space-time in relation to its surroundings) and intentional (a stone cannot deceive, resist or ignore); (c) deals with local unique meaningful events that are in relation with other unique events (life is psychologically meaningful in relation to birth and death). Living organisms in general possess the common attributes of hierarchical organization; dynamic flow of processes (an organism cannot stop living); and story (both individual and collective) (Von Bertalanffy 1952). So, psychic life is always *in relation to* something else, forming a whole (organism organization) as part of a whole (ecosystemic organization). This is of course not a new idea:

“*The conception of the system as a whole* as opposed to the *analytical* and *summative* points of view; the *dynamic conception* as opposed to the *static* and *machine-theoretical* conceptions; the consideration of the organism as a *primary activity* as opposed to the conception of its *primary reactivity*.” (Von Bertalanffy 1952, p. 19, *original italic*).

One of the most relevant results emerging from the analysis of psychology (Zagaria et al. 2020) is that current psychological sciences largely miss the systemic view, both in their methods and in the formation of their (confused) core concepts (Santana andLima 2020). One of the critical aspects of psychological sciences’ epistemology seems to be the “theoretical chaos” (Zagaria et al. 2020, p. ) in the many different definitions of some of its core concepts, such as “mind”, “cognition”, “emotion”, “consciousness”, etc. In the next paragraphs, I will try to argument that the problem is not in the heterogeneity of the definitions, rather in the way one forms the concepts.

## The Method of Complementary Negation

**Fig. 1:**
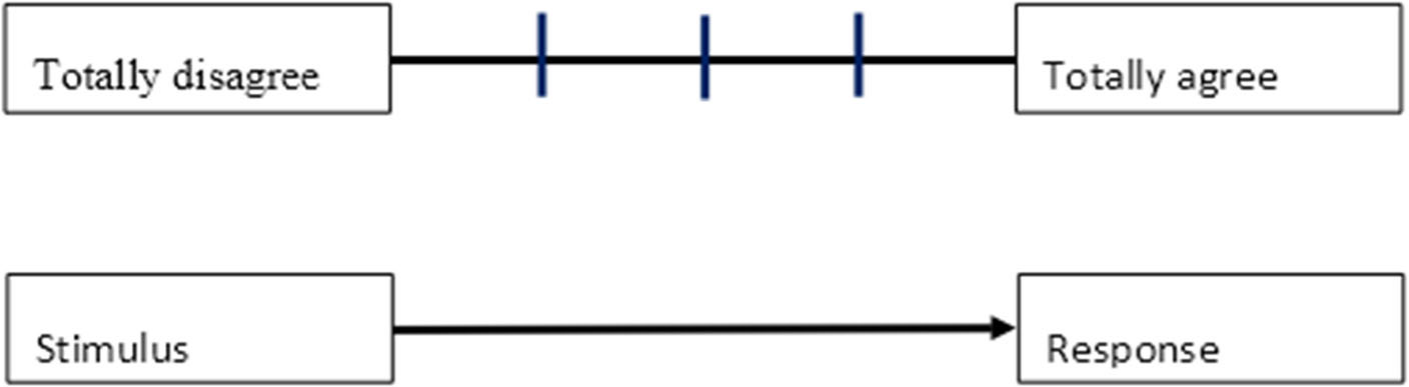
Examples of dyadic concepts in social sciences

The formation of concepts in social sciences is still characterized by dyadic structures, that is binaries: individualistic/collectivistic; qualitative/quantitative; marginal/mainstream; indigenous/universal; positive/negative; nature/nurture; stimulus/response; and so on (Baker 2009). Dyadic structures are resistant to change and do not leave space to developmental processes. Dyadic structures create non-hierarchical order of oppositions, whose steady state cannot be modified but by external events. For instance, by definition, the stimulus/response association is arbitrary to make possible any form of conditioning. Dyadic concepts are ubiquitous in social sciences, including psychology (Fig. 1).

**Fig. 2:**
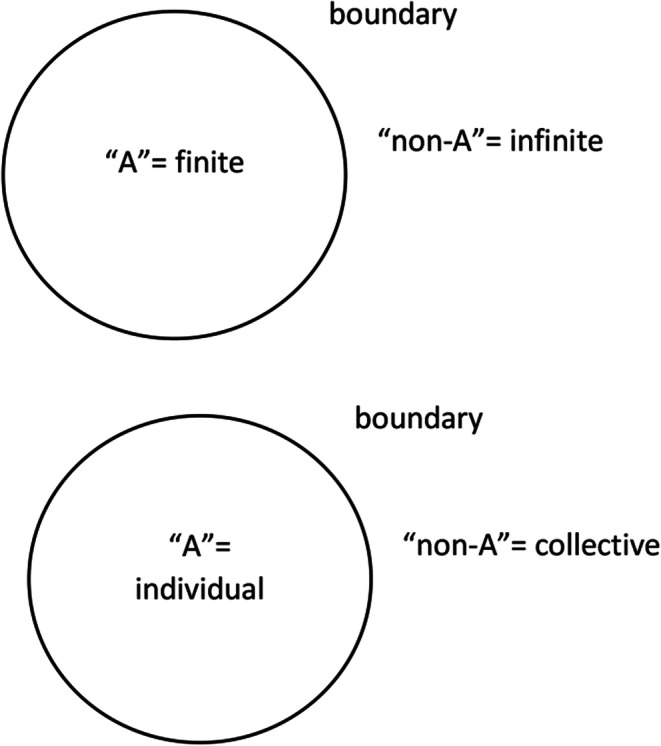
Triadic sets

Yet, thinking in binary terms creates a non-developmental form of concepts: “moving in any of these directions the transformations achieved remain contained within the logic of the given’’ (Herbst 1976, p. 29). The sociologist Herbst (1976) proposed to develop a different way of concept formation, based on what he called “behavioral logic”. The first axiom of his logic reads: ‘‘The primary conceptual unit is given as a triad of distinguishable undefined components, which are definable in terms of one another’’ (Herbst 1976, p. 90). It means that every time we create a concept, a triadic system emerges including the concept “A”, its logic negation “non-A”; and the boundary (Fig. 2).

**Fig. 3:**
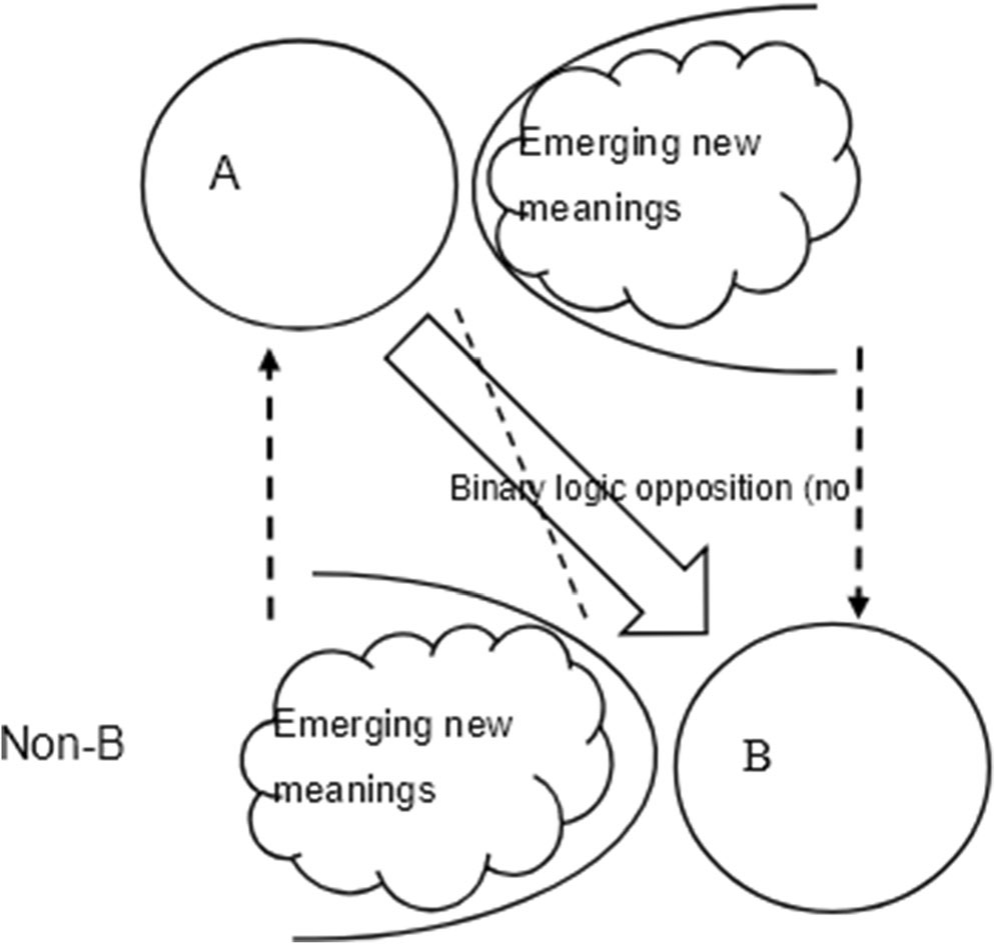
Cogenetic logic versus binary logic

Usually, concepts in social sciences are defined by creating dyadic systems of opposition between terms, as in the individual/collective example. But in logical terms, the negation of a concept is the non-concept, while the features of language lead us to define the negation in terms of a counter-concept, that is the concept that we oppose by habit, historical conditions or experience. So, the distinctions between nature and nurture, individual and collective, citizen and foreigner, is not logical rather conventional. For instance, the nature/nurture opposition emerged and became a naturalized binary category that blocked the development of new ideas, until the epigenetic perspective did not prove this opposition to be updated (Crews et al. 2014). The logical opposite of a category is not a different category, rather its logical negation “non-A” (Tateo 2016), which can be conventionally labeled in different ways according to the historical conditions. I suggest that the confusion in the conceptual system of psychology criticized by Zagaria et al. (2020) is not solved by simply establishing common core concepts definition in the form of dyadic structures. A brand-new way of concept formation is required, which I have called *method of complementary negation* (Tateo 2016). For every concept in social sciences, on must be able to account for the whole, composed by the triadic set [m, n, p] (‘‘A’’+‘‘non-A’’+‘‘boundary’). Each element of the triad is codefined by the others. Core concepts (e.g. culture, intelligence, emotion, etc.) imply the construction of a system of meaning in which one is able to define (a) the operation of primary distinction (the conditions to define a boundary), (b) the closed set A, and (c) the complementary open set non-A. (Fig. 3).

For instance, in order to have a productive use of the concept of “culture” in social sciences, one should be able to define its complementary set of what is “non-culture”. Usually, this is done by opposing the different category of “natural” but this is a logical fallacy. If one is not able to define at the same time *both* “culture” and “non-culture”, the former category is useless in practice. This is because it is not a dichotomy, rather a complementarity, that allows us to understand those manifold hybrid forms in which what is cultural and what is non-cultural form a whole, like in the case of gardening. This implies a metaphysical shift in which human beings are in mutually inclusive relationship with what is non-human (Rayner 2017).

This way of concept formation establishes a whole, an ecosystemic structure of concepts, in which the elements form a set of co-definitions. Such a system is also developmental to the extent that the tension established between the elements leads to the potential production of new meanings and to avoid tautological definitions that seem so common in psychology (Zagaria et al. 2020). Let’s take a as concrete example about one of the common concepts in psychology: intelligence, which seems ubiquitous in the history of the discipline. The method of complementary negation postulates that once one tries to define the concept, one must able to account also for its logical negation. So, one should be able to define what is “non-intelligence” and the conditions to produce the distinction. If one is not able to define “non-A”, then everything becomes “intelligence” and the concept itself loses any heuristic power. Moreover, it is important to note that the non-A set is potentially infinite including all the specimen of clear non-intelligence, but also all the intermediate forms of quasi-intelligence (emerging new meanings in Fig. 3), which can become potential candidates to be included (or excluded) in the “A” set as long as our knowledge expands (or restricts) the boundaries of the concept. Specimen that where not considered full forms of intelligence (e.g. some non-human phenomena) can later be included in the definition. This is the dynamic that makes the triadic logic system developmental. “A” is then definable only in relation to “non-A” once the conditions for the boundary are made explicit. If the boundary disappears, one is no longer able to define what is “non-A” the triad disappears and the concept “A” becomes useless. One can play some mental experiments with different concepts such as “culture”, “behaviour”, “mind”, evolution”, “action”, etc. The method of complementary negation is an intellectual exercise that deconstructs the naturalization of dyadic structure of concepts and that what one considers oppositions (e.g. clean < > dirty; public < > private) are actually complex relationships of meaning systems (Valsiner 2014). This would allow to overcome the idea of unification and generalization achieved by simply *not disagreeing* or finding *weak agreements* on core concepts of the discipline, as instead Zagaria et al. (2020) defend. The use of complementary negation method indeed implies the construction of concept-systems, in which also the relationships whole-parts must be understood. This avoids the risk of the overproduction of simple concept’s definitions that is so common in social science in general.

So far, I have presented an organismic view of the object of psychology (Von Bertalanffy 1952) and a systemic co-genetic logic of concept-formation (Tateo 2016) that I claim to be the starting point to overcome the useless controversy about the epistemic status of psychology, as presented in Zagaria et al. (2020). It is now time to complete my theoretical proposal by presenting an alternative view of the possible epistemic fundaments of psychological sciences: a third way in building an ecosystemic epistemology.

## Ecosystemic Epistemology

The image of human being that psychology tends to produce is that of a *golem*: a mechanism who is animated either by an inherited genetic program or by an acquired cultural program (Ingold 2000). Evolutionary psychology has added to this view the luring aura of evolutionary teleology: the current state of affair is the only possible because it results of evolution. When the environmental conditions are taken into account, they are understood as something that is *represented* (Ingold 2000) in the psychological structures, perpetuating a duality between organism and environment, or a mere *container* for the individuals.

Although “structure is the first thing that human mind looks for to explain the order of natural processes” (Von Bertalanffy 1952, p. 16), one cannot “consider structures as the primary basis of the vital order” (Von Bertalanffy 1952, p. 16). On the contrary, the organismic and ecological perspective provides us with a different view (Bateson 1973; Ingold 2000; Von Bertalanffy 1952). A living organism (as one assumes human beings to be) is characterized by a systemic organization, which is more than the mere sum of its parts: “a mutual interaction of the conditions present in the total system, by a *dynamic order* as we may call it.” (Von Bertalanffy 1952, p. 17, *original italic*) All the parts work together to “guarantee the maintenance, construction, restitution, and reproduction” of the system (Von Bertalanffy 1952, p. 13). The organism itself does not *live into*, *represents*, or *adapts to* the environment. Whilst the epistemology of evolutionary psychology becomes weak is exactly in the concept of *adaptation*, missing the point that human phylogenetic development is a process of *co-evolution* between different parts of local ecosystems. The organism as a whole rather *resonates* with its lifeworld (Gibson 1973). In order to understand an organism, one need to grasp not only “the morphological *hierarchy of parts*” but also the “*hierarchy of processes*” (Von Bertalanffy 1952, p. 42), in which the environment is not an independent variable but an interacting part. As science of the person, psychology should become aware that individuality is a relational concept, which is defined by the triad individual-non individual-boundary: “individuality is a limit which is approached but not reached, either in development or in evolution.” (Von Bertalanffy 1952, p. 49).

The epistemic development of psychology should thus be that of becoming a life science in the sense of an ecological science, an old idea which is as innovative as forgotten. Ecology is not the study of the individual *in* its environment, rather the study of the *local and unique ecosystemic configuration* of relationships. The adjective local.

“serves to indicate from where some people are speaking and acting. Therefore, it reveals the situation in space and time from which these populations express their knowledge, cultures, and their relationship with the environment. No one is omnipresent. Everyone, whether researcher or member of a traditional population, speaks from a specific viewpoint.” (Albuquerque andChaves Alves 2016, p. 5).

Ecology is the science of unique local phenomena whose laws can be generalized (Valsiner 2014). The forms and capacities of humans cannot be attributed to a linear process of evolution, an expression of genome independently from the local conditions, but to a *developmental system* (Ingold 2000), that is the *whole system of relations* constituted by the *presence* of the organism, including its genes, in a given environment. The object of psychology must then become a *whole of wholes* (Marsico andVarzi 2016).

The idea of evolution as a progressive selection of useful or fitting traits is inconsistent with the phenomenology of both human and non-human life. As Von Bertalanffy (1952) elegantly notes:

“In the living world the maxims ‘Why make it simple, when it can be complicated?’ and ‘It can be done this way, but it can also be done the other way’ seem to be much in vogue. Often astonishing roundabout ways are taken to reach a goal that could be reached far more simply and with less risk.” (p. 87).

It is exactly what one can observe in the terrific display of anthropodiversity: a number of local solutions to universal existential problems, such as giving birth, mating, dying, feeding, feeling pleasure, punishing, etc. Solutions are not always functional, economic or safe, the variety of human productions “in themselves have nothing to do with usefulness but can be fitted to different exigencies and habitats much in the same way as churches, town halls, or castles can be built equally well in gothic, baroque, or rococo styles.” (Von Bertalanffy 1952, p. 87) However, those manifold solutions are all *meaningful* in that local ecosystem.

Psychology should be neither a “hard” nor a “soft” science: it should become an ecological science, developing an ecosystemic epistemology, that considers the persons as complex organisms. The psychological processes must be considered as a whole also in their ontogenetic and sociogenetic development (Cole et al. 1978; Valsiner 2014; Vygotsky 1993, 1994).

Humans are themselves parts of local systems (including both human and non-human elements), as unique local configurations: the peculiar solutions to existential problems that one can call “culture”. These solutions include questions like “how do we organize family relationships” or “how do I differentiate myself from the other, but at the same time I do not appear an alien”. If one compares two or more local solutions in different places and times (even in evolutionary perspective), one would merely verify that people do some things in a similar way and some other things in a different way. Instead, considering the *developmental system* (Ingold 2000), one can appreciate how each individual constructs her personal trajectory of existence, negotiating the meaning of her life, the ambivalences and the personal variations of values and conducts. If the unification of psychology’s core knowledge is to be advocated, it can only be achieved by overcoming the old controversies and by developing an innovative epistemic ecological perspective.
